# *N*-[Bis(4-fluoro­phen­yl)methyl­ene]aniline

**DOI:** 10.1107/S1600536810007099

**Published:** 2010-03-03

**Authors:** Mei-Lin Zhang, Jie Yang

**Affiliations:** a College of Chemistry, Sichuan University, Chengdu 610064, People’s Republic of China; b Institute of Materials Science & Technology, Sichuan University, Chengdu 610064, People’s Republic of China

## Abstract

The title compound, C_19_H_13_F_2_N, was synthesized by an addition reaction of bis­(4-fluoro­phen­yl)methanone with aniline. The dihedral angles formed by the fluoro­benzene rings with the aniline ring are 81.04 (5) and 64.15 (5)°. In the crystal packing, inter­molecular C—H⋯F hydrogen bonds link mol­ecules into zigzag chains parallel to the *c* axis.

## Related literature

For synthetic applications of the title compound, see: Brink *et al.* (1993 ▶); Roovers *et al.* (1990 ▶). For the properties of deriv­atives of the title compound, see: Hedrick *et al.* (1993 ▶); Niswander & Martell (1978 ▶); Qi *et al.* (1999 ▶); Bourgeois *et al.* (1996 ▶).
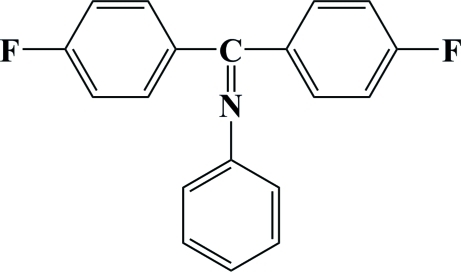


## Experimental

### 

#### Crystal data

C_19_H_13_F_2_N
*M* *_r_* = 293.30
Orthorhombic, 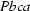

*a* = 18.104 (6) Å
*b* = 8.612 (3) Å
*c* = 18.985 (6) Å
*V* = 2960.0 (17) Å^3^
*Z* = 8
Mo *K*α radiation
μ = 0.09 mm^−1^
*T* = 293 K
0.40 × 0.27 × 0.11 mm

#### Data collection

Bruker SMART APEX CCD area-detector diffractometer
Absorption correction: multi-scan (*SADABS*; Bruker, 1996 ▶) *T*_min_ = 0.963, *T*_max_ = 0.990
13881 measured reflections
2612 independent reflections
2060 reflections with *I* > 2σ(*I*)
*R*_int_ = 0.022

#### Refinement

*R*[*F* ^2^ > 2σ(*F* ^2^)] = 0.035
*wR*(*F* ^2^) = 0.115
*S* = 0.98
2612 reflections
200 parameters
H-atom parameters constrained
Δρ_max_ = 0.15 e Å^−3^
Δρ_min_ = −0.17 e Å^−3^

### 

Data collection: *SMART* (Bruker, 1996 ▶); cell refinement: *SAINT* (Bruker, 1996 ▶); data reduction: *SAINT* program(s) used to solve structure: *SHELXS97* (Sheldrick, 2008 ▶); program(s) used to refine structure: *SHELXL97* (Sheldrick, 2008 ▶); molecular graphics: *SHELXTL* (Sheldrick, 2008 ▶); software used to prepare material for publication: *SHELXTL*.

## Supplementary Material

Crystal structure: contains datablocks global, I. DOI: 10.1107/S1600536810007099/rz2421sup1.cif

Structure factors: contains datablocks I. DOI: 10.1107/S1600536810007099/rz2421Isup2.hkl

Additional supplementary materials:  crystallographic information; 3D view; checkCIF report

## Figures and Tables

**Table 1:** Hydrogen-bond geometry (Å, °)

*D*—H⋯*A*	*D*—H	H⋯*A*	*D*⋯*A*	*D*—H⋯*A*
C18—H18⋯F1^i^	0.93	2.54	3.379 (2)	150

Symmetry code: (i) 
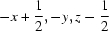
.

**Fig. 1.**
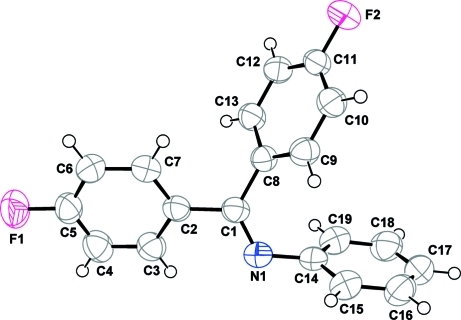
The molecular structure of title compound with displacement ellipsoids drawn at the 50° probability level.

## supplementary crystallographic information

### Comment

The title compound, *N*-[bis(4-fluorophenyl)methylene]aniline, can be used as the monomer of high performance polyarylene ether ketone (Brink *et al.*, 1993; Roovers *et al.*, 1990). Some derivatives of this compound have been reported with good thermostability and chemical-resistance (Hedrick *et al.*, 1993; Niswander & Martell, 1978; Qi *et al.*, 1999; Bourgeois *et al.*, 1996). We report here the crystal structure of the title compound.

In the molecule of the title compound (Fig. 1), the C1═N\ bond is 1.2839 (19) Å. The fluorobenzene rings form a dihedral angle of 66.52 (4)° and are oriented with respect to the aniline ring at dihedral angles of 81.04 (5) and 64.15 (5)°. In the crystal packing, intermolecular C—H···F hydrogen bonds (Table 1) link molecules into zig-zag chains parallel to the *c* axis.

### Experimental

General procedure for the synthesis of the title compound: bis(4-fluorophenyl)methanone (21.8 g, 0.10 mol), aniline (9.3 g, 0.10 mol), toluene (500 ml) and *p*-methylbenzenesulfonic acid (1.7 g, 0.01 mol) were charged into a three-necked round-bottomed flask fitted whith a mechanical stirrer, a nitrogen inlet and a thermometer. The mixture was stirred at 120°C for 2 h, then it was heated to boiling point and kept for 12 h under nitrogen atmosphere. After the reactor was cooled to room temperature, the reaction solution was poured into methanol. The resulting solid was filtered, washed with cold methanol, dried under vacuum to get yellow powder. Yellow crystals suitable for X-ray analysis were obtained by slow evaporation of a methanol solution at room temperature over a period a week.

### Refinement

All the H atoms could be found in the difference Fourier maps. They were positioned geometrically with C—H = 0.93 Å. *U*_iso_(H) = 1.2*U*_eq_ (aromatic C) while *U*_iso_(H) = 1.5*U*_eq_ (O).

### Crystal data

**Table:**

C_19_H_13_F_2_N	*F*(000) = 1216
*M**_r_* = 293.30	*D*_x_ = 1.316 Mg m^−^^3^
Orthorhombic, *P**b**c**a*	Mo *K*α radiation, λ = 0.71073 Å
Hall symbol: -P 2ac 2ab	Cell parameters from 1417 reflections
*a* = 18.104 (6) Å	θ = 2.4–24.1°
*b* = 8.612 (3) Å	µ = 0.09 mm^−^^1^
*c* = 18.985 (6) Å	*T* = 293 K
*V* = 2960.0 (17) Å^3^	Plate, yellow
*Z* = 8	0.40 × 0.27 × 0.11 mm

### Data collection

**Table:**

Bruker APEX CCD area-detector diffractometer	2612 independent reflections
Radiation source: fine-focus sealed tube	2060 reflections with *I* > 2σ(*I*)
graphite	*R*_int_ = 0.022
phi and ω scans	θ_max_ = 25.1°, θ_min_ = 2.2°
Absorption correction: multi-scan (*SADABS*; Bruker, 1996)	*h* = −16→21
*T*_min_ = 0.963, *T*_max_ = 0.990	*k* = −10→10
13881 measured reflections	*l* = −20→22

### Refinement

**Table:**

Refinement on *F*^2^	Secondary atom site location: difference Fourier map
Least-squares matrix: full	Hydrogen site location: inferred from neighbouring sites
*R*[*F*^2^ > 2σ(*F*^2^)] = 0.035	H-atom parameters constrained
*wR*(*F*^2^) = 0.115	*w* = 1/[σ^2^(*F*_o_^2^) + (0.0728*P*)^2^ + 0.3185*P*] where *P* = (*F*_o_^2^ + 2*F*_c_^2^)/3
*S* = 0.98	(Δ/σ)_max_ < 0.001
2612 reflections	Δρ_max_ = 0.15 e Å^−^^3^
200 parameters	Δρ_min_ = −0.17 e Å^−^^3^
0 restraints	Extinction correction: *SHELXL97* (Sheldrick, 2008), Fc^*^=kFc[1+0.001xFc^2^λ^3^/sin(2θ)]^-1/4^
Primary atom site location: structure-invariant direct methods	Extinction coefficient: 0.0017 (5)

### Special details

**Table:**

Geometry. All esds (except the esd in the dihedral angle between two l.s. planes) are estimated using the full covariance matrix. The cell esds are taken into account individually in the estimation of esds in distances, angles and torsion angles; correlations between esds in cell parameters are only used when they are defined by crystal symmetry. An approximate (isotropic) treatment of cell esds is used for estimating esds involving l.s. planes.
Refinement. Refinement of *F*^2^ against ALL reflections. The weighted *R*-factor wR and goodness of fit *S* are based on *F*^2^, conventional *R*-factors *R* are based on *F*, with *F* set to zero for negative *F*^2^. The threshold expression of *F*^2^ > σ(*F*^2^) is used only for calculating *R*-factors(gt) etc. and is not relevant to the choice of reflections for refinement. *R*-factors based on *F*^2^ are statistically about twice as large as those based on *F*, and *R*- factors based on ALL data will be even larger.

### Fractional atomic coordinates and isotropic or equivalent isotropic displacement parameters (Å^2^)

**Table:**

	*x*	*y*	*z*	*U*_iso_*/*U*_eq_	
C1	0.20644 (7)	0.24801 (15)	0.27677 (8)	0.0490 (3)	
C2	0.26143 (8)	0.25396 (15)	0.33496 (8)	0.0505 (4)	
C3	0.33329 (8)	0.20396 (17)	0.32292 (9)	0.0591 (4)	
H3	0.3463	0.1672	0.2786	0.071*	
C4	0.38557 (10)	0.20799 (19)	0.37563 (10)	0.0676 (5)	
H4	0.4335	0.1733	0.3675	0.081*	
C5	0.36536 (9)	0.2642 (2)	0.44019 (9)	0.0645 (4)	
C6	0.29542 (9)	0.3134 (2)	0.45459 (9)	0.0683 (5)	
H6	0.2831	0.3498	0.4992	0.082*	
C7	0.24335 (9)	0.30831 (19)	0.40158 (8)	0.0608 (4)	
H7	0.1954	0.3418	0.4106	0.073*	
C8	0.12682 (7)	0.23425 (15)	0.29640 (7)	0.0463 (3)	
C9	0.07420 (8)	0.33442 (17)	0.26868 (8)	0.0538 (4)	
H9	0.0887	0.4115	0.2373	0.065*	
C10	0.00099 (8)	0.32128 (18)	0.28699 (8)	0.0576 (4)	
H10	−0.0340	0.3894	0.2688	0.069*	
C11	−0.01927 (8)	0.20545 (17)	0.33273 (8)	0.0527 (4)	
C12	0.03040 (8)	0.10401 (17)	0.36102 (8)	0.0559 (4)	
H12	0.0151	0.0259	0.3916	0.067*	
C13	0.10393 (8)	0.12022 (16)	0.34313 (8)	0.0533 (4)	
H13	0.1387	0.0535	0.3628	0.064*	
C14	0.18353 (8)	0.22899 (17)	0.15481 (8)	0.0531 (4)	
C15	0.16862 (9)	0.35005 (19)	0.10865 (8)	0.0634 (4)	
H15	0.1886	0.4479	0.1168	0.076*	
C16	0.12419 (10)	0.3250 (2)	0.05081 (9)	0.0710 (5)	
H16	0.1133	0.4069	0.0207	0.085*	
C17	0.09587 (10)	0.1799 (2)	0.03724 (9)	0.0722 (5)	
H17	0.0659	0.1638	−0.0018	0.087*	
C18	0.11214 (10)	0.0590 (2)	0.08170 (9)	0.0725 (5)	
H18	0.0936	−0.0395	0.0722	0.087*	
C19	0.15577 (9)	0.08234 (19)	0.14032 (9)	0.0640 (4)	
H19	0.1666	−0.0003	0.1701	0.077*	
F1	0.41742 (6)	0.27364 (14)	0.49157 (6)	0.0917 (4)	
F2	−0.09151 (5)	0.19054 (12)	0.34959 (5)	0.0732 (3)	
N1	0.23085 (7)	0.25324 (14)	0.21332 (7)	0.0575 (3)	

### Atomic displacement parameters (Å^2^)

**Table:**

	*U*^11^	*U*^22^	*U*^33^	*U*^12^	*U*^13^	*U*^23^
C1	0.0464 (8)	0.0492 (7)	0.0515 (9)	−0.0010 (6)	0.0039 (6)	0.0047 (6)
C2	0.0473 (8)	0.0524 (7)	0.0519 (9)	−0.0050 (6)	0.0023 (6)	0.0062 (6)
C3	0.0527 (9)	0.0633 (9)	0.0614 (10)	0.0036 (7)	0.0011 (7)	0.0018 (7)
C4	0.0535 (9)	0.0703 (10)	0.0790 (13)	0.0052 (7)	−0.0069 (9)	0.0084 (8)
C5	0.0616 (10)	0.0718 (10)	0.0601 (11)	−0.0123 (8)	−0.0132 (8)	0.0184 (8)
C6	0.0669 (11)	0.0877 (11)	0.0504 (9)	−0.0179 (8)	0.0009 (8)	0.0038 (8)
C7	0.0505 (8)	0.0762 (10)	0.0556 (9)	−0.0082 (7)	0.0055 (7)	0.0003 (8)
C8	0.0459 (7)	0.0475 (7)	0.0455 (8)	−0.0007 (6)	0.0010 (6)	−0.0008 (6)
C9	0.0568 (9)	0.0515 (8)	0.0531 (9)	0.0033 (6)	0.0037 (7)	0.0069 (6)
C10	0.0509 (8)	0.0627 (8)	0.0591 (9)	0.0107 (7)	0.0008 (7)	0.0027 (7)
C11	0.0418 (7)	0.0652 (9)	0.0513 (9)	−0.0038 (6)	0.0033 (6)	−0.0098 (7)
C12	0.0544 (8)	0.0589 (8)	0.0543 (9)	−0.0093 (7)	0.0028 (7)	0.0064 (7)
C13	0.0484 (8)	0.0543 (8)	0.0570 (9)	0.0006 (6)	−0.0013 (7)	0.0088 (7)
C14	0.0452 (7)	0.0679 (9)	0.0463 (8)	0.0012 (6)	0.0099 (6)	0.0013 (7)
C15	0.0685 (10)	0.0643 (9)	0.0574 (10)	−0.0017 (7)	0.0046 (8)	0.0061 (8)
C16	0.0723 (11)	0.0837 (12)	0.0571 (10)	0.0078 (9)	0.0012 (9)	0.0122 (9)
C17	0.0640 (10)	0.0992 (13)	0.0534 (10)	−0.0021 (9)	0.0007 (8)	−0.0032 (9)
C18	0.0776 (11)	0.0758 (11)	0.0640 (11)	−0.0118 (9)	0.0051 (9)	−0.0085 (9)
C19	0.0695 (10)	0.0636 (9)	0.0589 (10)	0.0008 (8)	0.0060 (8)	0.0047 (7)
F1	0.0796 (7)	0.1177 (9)	0.0777 (8)	−0.0138 (6)	−0.0290 (6)	0.0189 (6)
F2	0.0456 (5)	0.0960 (7)	0.0778 (7)	−0.0052 (4)	0.0076 (4)	−0.0007 (5)
N1	0.0497 (7)	0.0713 (8)	0.0514 (8)	−0.0028 (6)	0.0052 (6)	0.0058 (6)

### Geometric parameters (Å, °)

**Table:**

C1—N1	1.2839 (19)	C10—H10	0.9300
C1—C2	1.488 (2)	C11—F2	1.3527 (17)
C1—C8	1.494 (2)	C11—C12	1.364 (2)
C2—C7	1.388 (2)	C12—C13	1.381 (2)
C2—C3	1.389 (2)	C12—H12	0.9300
C3—C4	1.378 (2)	C13—H13	0.9300
C3—H3	0.9300	C14—C15	1.388 (2)
C4—C5	1.368 (3)	C14—C19	1.387 (2)
C4—H4	0.9300	C14—N1	1.418 (2)
C5—F1	1.3589 (19)	C15—C16	1.378 (2)
C5—C6	1.363 (3)	C15—H15	0.9300
C6—C7	1.380 (2)	C16—C17	1.375 (3)
C6—H6	0.9300	C16—H16	0.9300
C7—H7	0.9300	C17—C18	1.373 (3)
C8—C13	1.3868 (19)	C17—H17	0.9300
C8—C9	1.389 (2)	C18—C19	1.379 (2)
C9—C10	1.375 (2)	C18—H18	0.9300
C9—H9	0.9300	C19—H19	0.9300
C10—C11	1.372 (2)		
			
N1—C1—C2	117.71 (13)	C11—C10—H10	120.7
N1—C1—C8	124.69 (13)	F2—C11—C12	118.91 (14)
C2—C1—C8	117.59 (12)	F2—C11—C10	118.50 (13)
C7—C2—C3	118.38 (15)	C12—C11—C10	122.59 (14)
C7—C2—C1	122.04 (14)	C11—C12—C13	118.29 (14)
C3—C2—C1	119.58 (14)	C11—C12—H12	120.9
C4—C3—C2	121.07 (16)	C13—C12—H12	120.9
C4—C3—H3	119.5	C12—C13—C8	121.13 (13)
C2—C3—H3	119.5	C12—C13—H13	119.4
C5—C4—C3	118.42 (16)	C8—C13—H13	119.4
C5—C4—H4	120.8	C15—C14—C19	119.22 (15)
C3—C4—H4	120.8	C15—C14—N1	120.08 (14)
F1—C5—C6	118.79 (17)	C19—C14—N1	120.55 (14)
F1—C5—C4	118.62 (16)	C16—C15—C14	119.91 (16)
C6—C5—C4	122.58 (16)	C16—C15—H15	120.0
C5—C6—C7	118.58 (17)	C14—C15—H15	120.0
C5—C6—H6	120.7	C17—C16—C15	120.63 (17)
C7—C6—H6	120.7	C17—C16—H16	119.7
C6—C7—C2	120.97 (16)	C15—C16—H16	119.7
C6—C7—H7	119.5	C16—C17—C18	119.62 (17)
C2—C7—H7	119.5	C16—C17—H17	120.2
C13—C8—C9	118.51 (13)	C18—C17—H17	120.2
C13—C8—C1	120.28 (12)	C19—C18—C17	120.55 (17)
C9—C8—C1	121.21 (13)	C19—C18—H18	119.7
C10—C9—C8	120.95 (14)	C17—C18—H18	119.7
C10—C9—H9	119.5	C18—C19—C14	120.02 (16)
C8—C9—H9	119.5	C18—C19—H19	120.0
C9—C10—C11	118.51 (13)	C14—C19—H19	120.0
C9—C10—H10	120.7	C1—N1—C14	121.45 (12)

### Hydrogen-bond geometry (Å, °)

**Table:**

*D*—H···*A*	*D*—H	H···*A*	*D*···*A*	*D*—H···*A*
C18—H18···F1^i^	0.93	2.54	3.379 (2)	150

Symmetry codes: (i) −*x*+1/2, −*y*, *z*−1/2.
